# Expanding the coverage and accuracy of parcel-level land value estimates

**DOI:** 10.1371/journal.pone.0291182

**Published:** 2023-09-08

**Authors:** Miriam Gold, Seth Binder, Christoph Nolte

**Affiliations:** 1 Department of Economics and Department of Environmental Studies, St. Olaf College, Northfield, Minnesota, United States of America; 2 Department of Earth & Environment, Boston University, Boston, Massachusetts, United States of America; Gebze Teknik Universitesi, TURKEY

## Abstract

Planning for cost-effective conservation requires reliable estimates of land costs, spatially-differentiated at high resolution. Nolte (2020) provides a county-by-county, parcel-level estimation approach that dramatically improves estimates of fair market value for undeveloped land across the contiguous Unites States. Much undeveloped land of conservation interest is under threat of conversion to agricultural use or is already agricultural. This paper demonstrates the value of accounting for additional variables that affect agricultural productivity and demand for undeveloped land, as well as the benefit of modeling at scales corresponding to regional agricultural markets. We find that countywide median home value, climatic variables, and several parcel-level soil type variables contribute substantially to predictive power. Enlarging the set of predictors and the geographical scale of modeling improves accuracy by approximately 15 percent and, relative to a more restricted modeling benchmark adapted from Nolte (2020), extends coverage into 376 counties occupying 1.35 million km^2^. To assess the practical benefits of our modeling approach, we simulate the protection of 30 percent of US lands via government purchasing, modeled after the Biden administration’s “30x30” initiative. Using our proposed modeling strategy, the purchasing agency saves approximately $15 million per year, or 4 percent of the USDA’s annual land easement budget.

## Introduction

Agencies and private organizations designing conservation efforts must contend with the fundamental scarcity of available resources, while simultaneously confronting a vast array of potential conservation strategies. Accurately predicting costs of competing strategies, then, is crucial to achieving the cost-effective use of scarce conservation dollars. Where conservation by fee simple acquisitions and land easement contracts is the dominant approach, the market value of land is a primary determinant of such costs. Yet, academics and planners have not often had access to reliable estimates of fair market land value, which constitute a high-priority resource for global change science and policy in general [1]. Some studies of conservation cost have relied on state or county-wide average land value estimates (e.g., [2, 3]), which obscure important heterogeneity of land values across those geographically large political units. Recent work suggests that conservation strategies based on such low-resolution cost estimates can substantially underestimate the cost of selected plans due to the unobserved heterogeneity in land values [4].

Several studies have attempted more accurate, higher-resolution land value estimates with national coverage. Larson (2015) uses a patchwork of USDA county-level agricultural land value estimates and existing hedonic estimates of urban land value [5] to interpolate values to a mix of parcels, census tracts, and counties across the contiguous U.S. [6]. Utilizing mortgage appraisal data, Davis et al. (2021) estimate residential land values nationwide at sub-county resolution (zip codes and census tracts) [7]. Albouy et al. (2018) estimate urban land values for every Metropolitan Statistical Area (MSA) in the U.S. [8]. For each MSA, they leverage sales data from the CoStar COMPS database to estimate land value per acre as a continuous function of distance from the city center. The majority of conservation interest, of course, lies outside of urban areas. Wentland et al. (2020) use microdata from the real estate marketplace firm Zillow, land attributes, and hedonic price modeling to produce bottom-up national land value accounts at the regional level [9]. With a primary focus on undeveloped land, Nolte (2020) integrates a nationwide database of parcel-specific information, including location, built environment, local demographics, and physical landscape features, with Zillow’s ZTRAX database of millions of sales records for use in a machine-learning algorithm to estimate land values at much higher spatial resolution [4]. These estimates explain a much greater portion of observed variation in sales value than county-wide averages. The Nolte approach constitutes a leap forward for estimating the value of undeveloped land value for conservation planning. As we show here, it can be further improved.

There is reason to believe that expanding both the set of agriculturally and economically relevant predictors as well as the geographic scale of analysis could improve the coverage and quality of land value estimates. The market value of undeveloped land is in many cases driven by the value of its actual or potential use in agriculture. It can also be driven by demand in related, residential land markets. Climate and soil variables, absent from the set of predictors in Nolte [4], are well known to be important determinants of agricultural productivity and land value. Irrigation can also be an important determinant of productivity for particular crops and geographies, though it requires costly investment. The irrigation status of land can indicate unobserved investments in machinery, infrastructure or physical changes to land that enhance its market value.

While many of the land quality attributes that determine economic value are fixed, or change only very slowly over time, many drivers of supply and demand—income, preferences, technology, and the availability of substitutes—vary over time. Failing to account for time-varying predictors can lead to an erosion of predictive power. When sales prices exhibit trends over time, not only will overall estimation accuracy suffer, but models might systematically over- or under-predict sales values for observations before or after the median sales year if they do not adequately control for such trends. To address these issues, we add median home price indices to our set of predictors. Home price indices capture changing dynamics in residential land markets that can exert pressure on (or otherwise correlate with) the price of undeveloped land. Accounting for variation in local home prices might thus improve the predictive power of models aimed at estimating the value of agricultural or other undeveloped land.

Beyond incorporating additional predictors, we consider the possibility that modeling at scales larger than the county can ease local observation constraints, improving both the coverage and accuracy of resulting estimates. Many counties have few sales records in the ZTRAX database. Nolte (2020) implements a 1000-observation minimum for modeling parcel land values within a county, supplementing a focal county’s observations with those drawn from neighboring counties where possible. In replicating this supplementation procedure with vacant, undeveloped, and agricultural parcels, we find that 27 percent of counties in the study sample fail to meet the threshold, and prediction accuracies for counties with sufficient-but-low observation densities are notably poorer than those for other counties.

**Fig 1:**
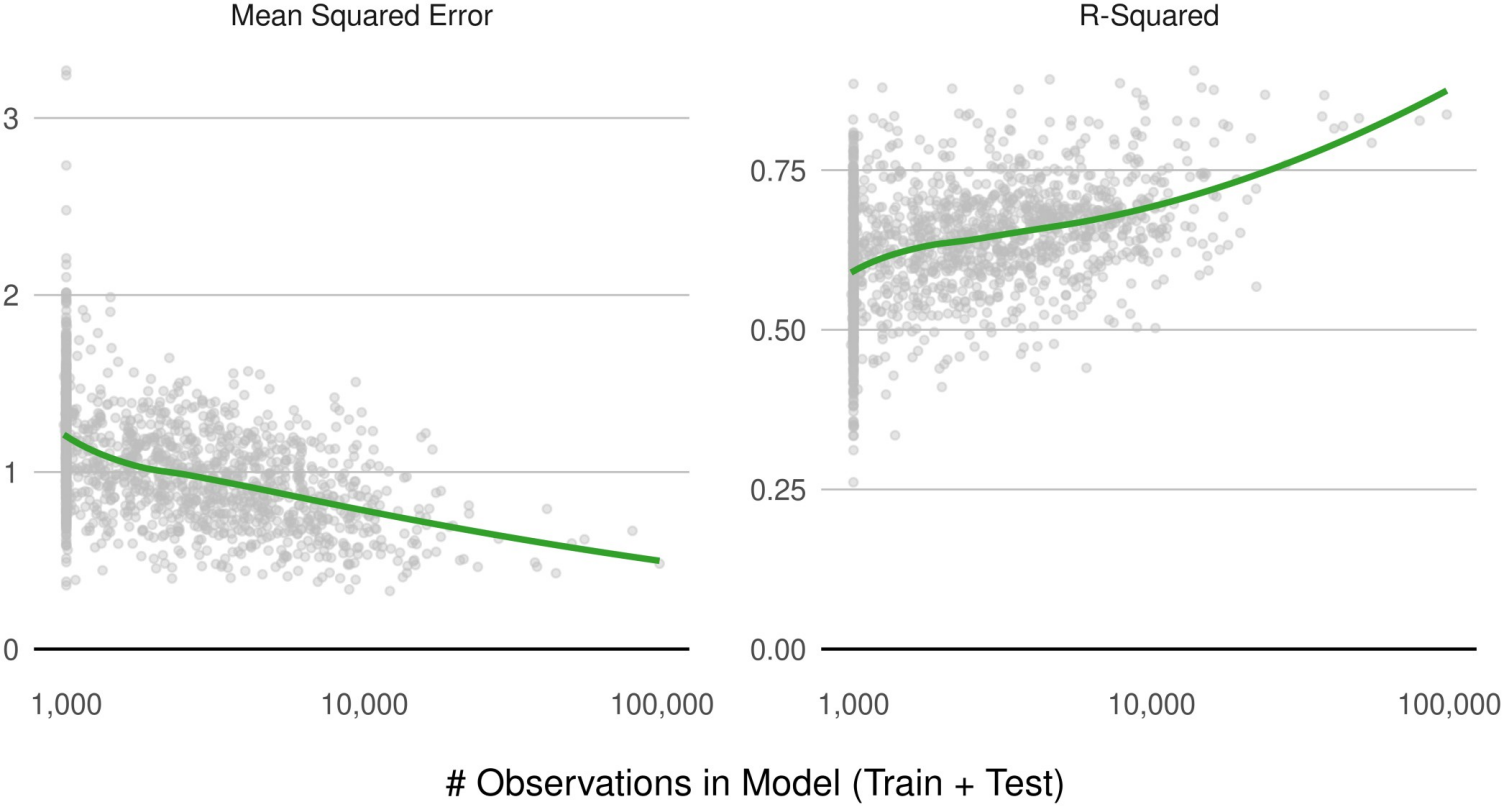
Model performance and sample size. Performance of county-level models using the baseline (“Restricted”) predictor set, plotted against the number of county-level observations. Each county in the analysis is represented by a gray circle. Green curves depict trend lines estimated using the non-parametric LOESS smoother in **R**.

Indeed, national performance aggregations mask substantial heterogeneity across county characteristics. In particular, a county’s observation density is strongly correlated with both performance measures. Fig 1 shows the relationship between model performance and the total number of observations in the county where the model was trained and tested. The monotonicity of the decrease (increase) in mean squared error (r-squared) as county-level models increase in size affirms the intuitive benefits of greater sample sizes. In pursuit of larger datasets, however, analysts will encounter a fundamental challenge: expanding the sample size necessarily broadens the geographic range from which observations will be drawn. This may debilitate the model’s predictive power if the broader geographic scope mixes land markets with disparate characteristics, economic dynamics, and time trends. Yet failing to expand the geographic scope has its own costs. In states such as Wyoming and Idaho, any given county has too few observations to model land value, forcing the analyst to discard potentially useful data.

**Fig 2:**
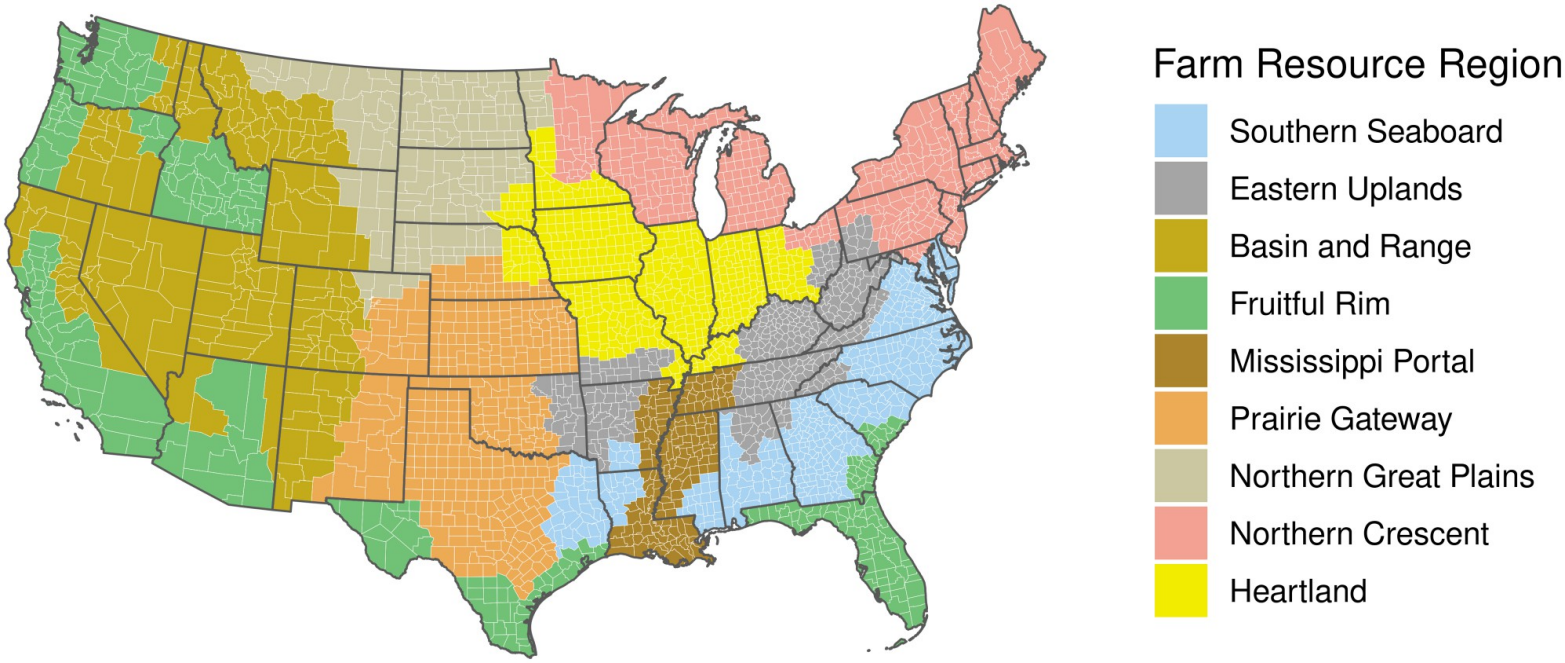
USDA farm resource regions. USDA assigns each county, in its entirety, to one of the nine farm resource regions shown here. County borders are demarcated with white lines.

We attempt to resolve this trade-off between model size and observation homogeneity by specifying models at the scale of USDA farm resource regions (Fig 2). Farm resource regions (FRR) are delineated in consideration of county-level, often cross-state similarities in geography and agricultural production [10].

Overall, our modeling approach, which both expands the set of predictors and models at a regional scale, reduces prediction error (MSE) by 15 percent. Decomposing this effect, we see that adding variables alone (without modeling at a regional scale) reduces prediction error by approximately 9 percent, while modeling at a regional scale *further* reduces prediction error by 5 percent (based on common parcels modeled at both scales, using the full set of predictors). By modeling at the farm resource region level, we extend coverage in areas that were dropped in county modeling due to insufficient density by 376 counties while preserving comparable predictive accuracy to county-level approaches.

In addition, we assess the practical benefits of our modeling approach by simulating the protection of 30 percent of US lands via government purchasing, modeled after the Biden administration’s “30x30” initiative. Using our proposed modeling strategy saves the purchasing agency approximately $15 million per year, or 4 percent of the USDA’s annual budget for land easements (a conservation strategy related to but distinct from direct land acquisition wherein property does not change hands but does become subject to certain land use restrictions and stewardship mandates). The provision of more accurate *ex ante* land value predictions can improve conservation outcomes by helping agencies achieve better targeted, more cost-effective contracting.

In short, our approach enhances the quality and quantity of parcel-level estimates of fair market value.

## Materials and methods

**Fig 3:**
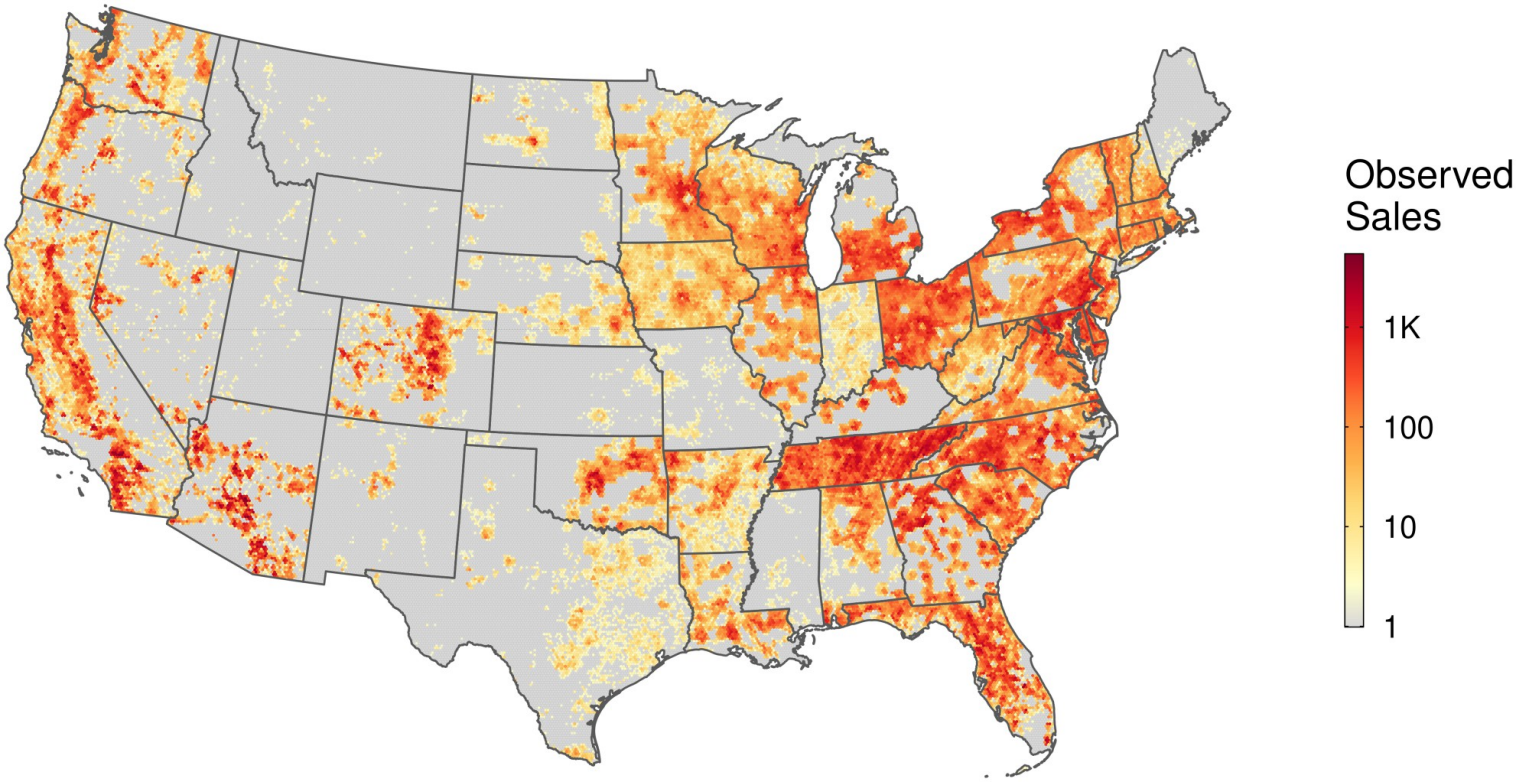
Spatial distribution of sales observations. A heat map of ZTRAX sales observations (total *N* = 5.04 million) in our filtered dataset for the sample period 2000–2019. Cells represent 10 km^2^ tessellation of the conterminous US.

We base our analysis on the approach first published by Nolte (2020) [4], which trains a tree-based, ensemble learning algorithm on a comprehensive high-resolution dataset of parcel characteristics and sales across the contiguous United States (140.9 million properties across 3,055 counties) to estimate the logged, inflation-adjusted, per-hectare value of parcels lacking recent sales data. The present study uses extremely randomized trees, a decision tree-based bagging algorithm, with 500 base learners (trees), $\frac{p}{3}$ random features tried at each split, where *p* is the total set of predictive features, and a required minimum leaf size of 3. Nolte (2020) estimates models for the group of all parcels greater than 1 acre in size as well as separate models for the subset of stringently defined “vacant” (undeveloped) parcels. Parcels of interest in the present study include all “vacant” parcels plus any additional parcels coded as agricultural, even if they contain a building footprint. Our filter (fully described in S1 Appendix) results in 5.04 million unique sales observations nationwide for the period 2000–2019, split into testing (*n = 1.26 million*) and training (*n = 3.78 million*) sets balanced on the outcome variable (Fig 3). In county models, this 0.75:0.25 split occurs within the county; in farm resource region models, training and testing observations are distributed farm resource region-wide. For county-level models, counties with fewer than 1000 observations are augmented with randomly sampled sales from adjacent counties until the focal county reaches 1000 observations. If the county and its neighbors combined fail to achieve 1000 complete observations, no model is specified.

Our approach to dealing with insufficient county-level observations differs in two ways from that of Nolte (2020). First, Nolte (2020) accepts donations from all counties, beginning with those nearest the focal county and extending the search outward until 1000 observations are achieved. We suspend the search if the directly adjacent neighbors of the focal county cannot achieve 1000 observations. Second, we supply the focal county with exactly the difference between its native sample size and 1000, whereas Nolte (2020) donates the entirety of the sample from the neighboring county, regardless of the neighboring county’s sample size. Further, because we restrict the generation of predictions to within each modeled county, our “base” model (i.e., Restricted predictor set, county level) does not produce predictions for all counties in CONUS, unlike Nolte (2020), which estimates land values for all parcels, even if that requires the utilization of a model specified in a distant county. Our county-level modeling approach allows for the specification of 1571 county models. In speaking of the extension in prediction geography our region-scale modeling offers, this approach is the benchmark to which we compare the results of our farm resource region models.

We restrict the benchmark modeling approach to each focal county and its immediately adjacent neighbors to better preserve the value of specifying models at the county level: the ability to capture locally specific dynamics. If county modeling did, in fact, perform better than modeling at the regional-scale, then this restricted approach would better evince such performance by not eroding the improvements from locally specific relationships by importing observations from great distances. Further, in counties with no sales observations, model validation is infeasible (Fig 1), hindering like-for-like model comparisons across geographic scales.

Among the set of predictors in Nolte (2020) [4] are variables on building presence, development, accessibility, local demographics, local nature preservation, terrain, water, land cover type, location, and date of sale. To these base data, we add variables for climate, irrigation status, soil quality, and local real estate prices. (Our predictor set omits two flood risk variables used in Nolte (2020).) Our climate variables are 30-year, climate normals of monthly minimum, mean, and maximum temperature, as well as dew temperature and precipitation from the PRISM Climate Group [11]. We aggregate these to the meteorological season. Parcel irrigation status is based on annual 1997–2017 estimates at a resolution of 30 sq. meters, produced by the Landsat-based Irrigation Dataset, or LANID-US [12]. We create two binary irrigation variables to record 1) whether the parcel had ever been irrigated prior to sale, and 2) whether it was irrigated at any point in the 3 years immediately preceding the sale.

Soil classifications, indicating a map unit’s suitability for agricultural use, were compiled from the Natural Resources Conservation Service (NRCS)’s high-resolution SSURGO soil survey database [13]. The NRCS soil survey provides map unit polygons that describe soil components (e.g., “loamy fine sand, 0 to 2 percent slopes”) and characteristics, including water capacity, flooding frequency, farmland classification, and features limiting development, among others. Soil farmland classifications, indicating a map unit’s suitability for agriculture, fall under five general classes, defined by the USDA:

1. **Prime**. Optimal site composition and availability for producing agricultural product;
2. **Unique**. Soil producing high-value crops (e.g., vineyards in California);
3. **Statewide Importance**. State-defined agricultural land that fails to meet prime criteria;
4. **Local importance**. Locally defined agricultural land that fails to meet prime or statewide criteria
5. **Conditional classes**. Land that would be considered prime, of statewide importance, or of local importance conditional on a specified improvement (e.g., “Prime if drained and protected from flooding”)

We operationalize farmland classifications as the percent of each parcel containing a given classification (e.g., “Prime Farmland” or “Farmland of Statewide Importance”). Aggregation is applied to overlapping classifications containing multiple conditions. For instance, “Prime if drained or protected from flooding” gets assigned to “Prime if drained” and “Prime if protected from flooding.” For sales records covering multiple parcels, our soil aggregation strategy is described in the S2 Appendix.

To incorporate information on local real estate markets, we add yearly all-transaction house price index values at the county level [14]. At the farm resource region level, to allow for comparability between counties, we source open access home value data from Realtor.com [15], which provides monthly county-level reports of median listing price from July 2016 to present. We select 2017 as the base year due to it having the lowest proportion of data quality flags (an automatically triggered indicator of prices being outside their normal range) of any sample year. Median listing prices are summarized at the year-county level and then multiplied by corresponding HPI values (with base year 2017) to estimate dollar values across the entire 2000–2020 sample period. In counties missing HPI values, median home value is estimated as the inverse squared distance weighted mean of estimated median home values in every county within 150 km of the focal county.

Altogether, we estimate fair market value in four primary model runs, corresponding to different combinations of predictor set (our “Full” set of predictors or the “Restricted” set including only variables from Nolte (2020)) and geographic scale of analysis (county-level or farm resource region-level). **R** code for all of the methods described above is available at https://github.com/binders1/fmv.

## Results

### Predicted fair market land value

**Fig 4:**
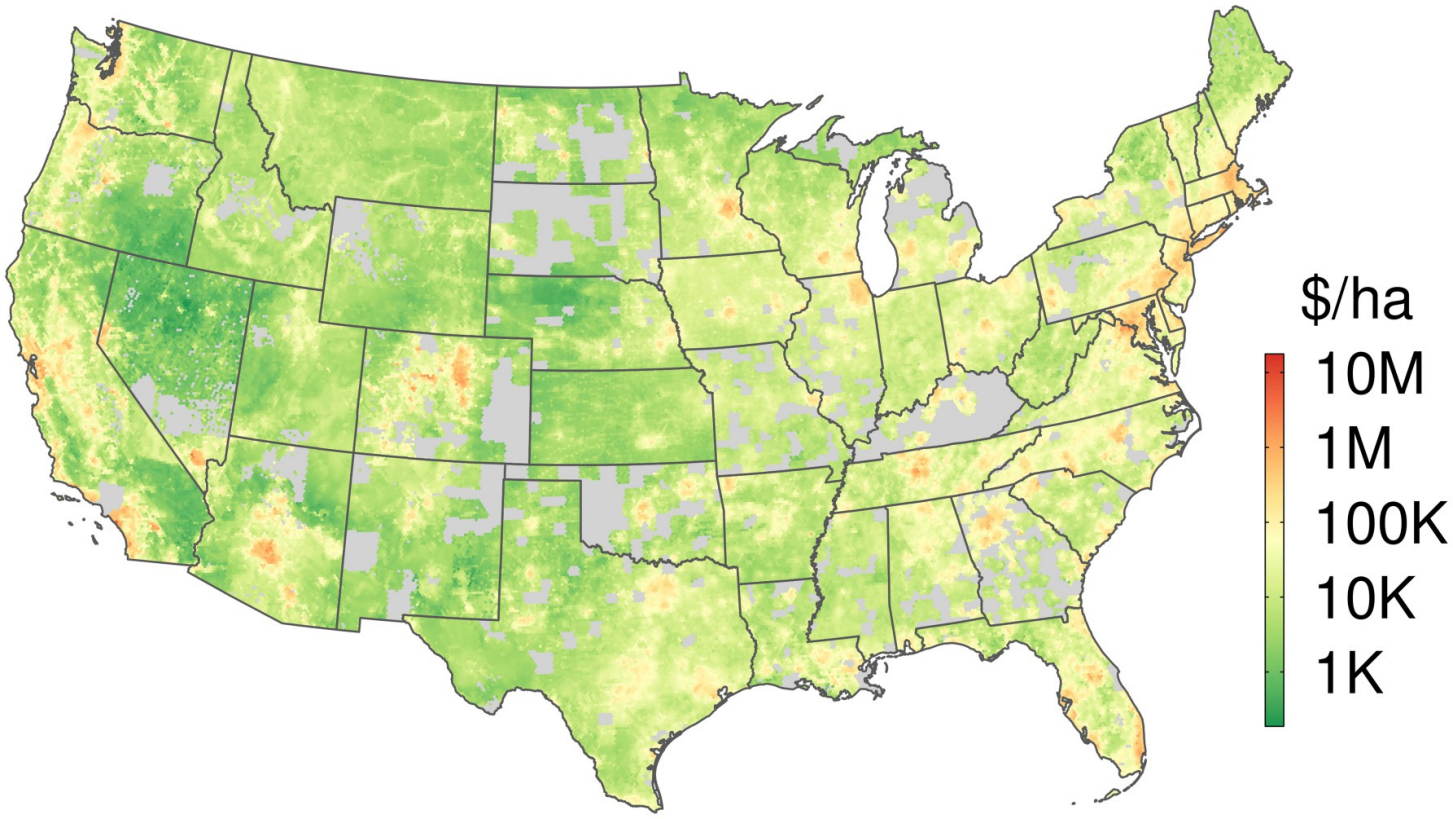
Estimated land values. Fair market land values (in 2020 USD per hectare), shown at 10 km^2^ resolution, based on predictions for 31.35 million parcels generated by the building-free model estimated at the scale of farm resource regions. Gray portions of the map indicate areas for which we lack adequate parcel data.

To assess our model’s comparability to the results of Nolte (2020), we predict the year-2020 fair market land value of all 31.35 million parcels in our base dataset, including those for which we do not have sales records (Fig 4). The predictions are generated using a model built at the farm resource region-level, using the full predictor set. We exclude parcels with a building footprint from the training dataset, so that model predictions may be interpreted as unimproved land value.

Our results largely replicate geographic patterns in Nolte (2020)’s CONUS-wide prediction map. Per-hectare values greatly increase in metropolitan areas, with the urban centers of Washington D.C., Boston, and Los Angeles reaching predicted prices of well over $1 million. Because the model predicts land value alone, not including built improvements, we observe a gentler gradient between rural and urban regions than is shown in Nolte (2020)’s Fig 1. This gradient is further smoothed visually by our mapping method, which averages predicted values over 10 km^2^ hexagonal tessellation cells.

While the geographic pattern and general order of magnitude of our fair market value estimates roughly match the Nolte (2020) results, our added predictors and regional modeling strategy deliver important improvements in accuracy and coverage.

### The effect of added predictors

**Fig 5:**
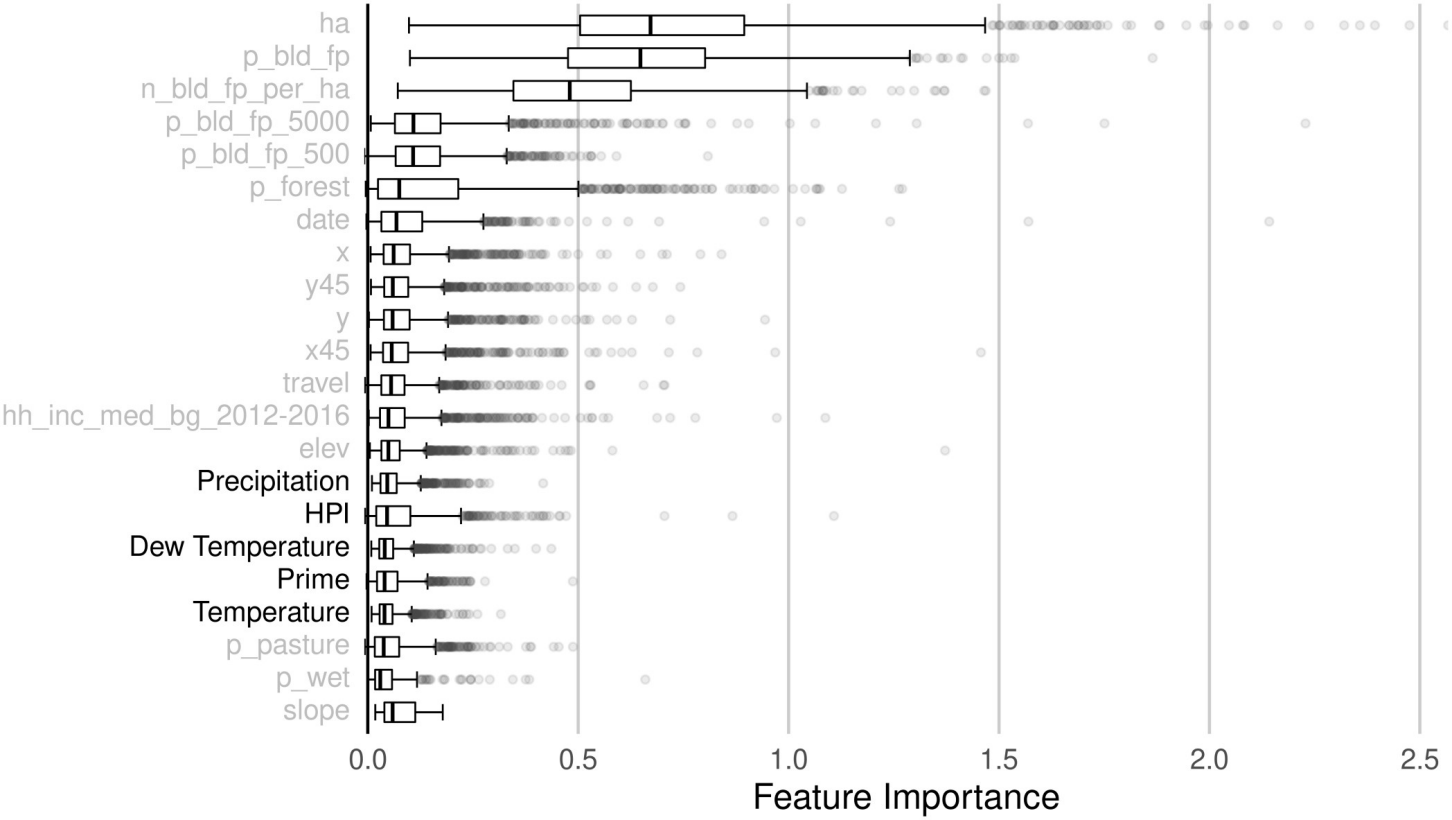
Permutation feature importance, county. Box plots of permutation feature importance for the top 20 predictors, across all county-level models estimated using the full predictor set. Predictors added by this paper are denoted in bold, and climatic variables have been grouped for clarity. Gray circles represent outlier county values for each predictor.

**Fig 6:**
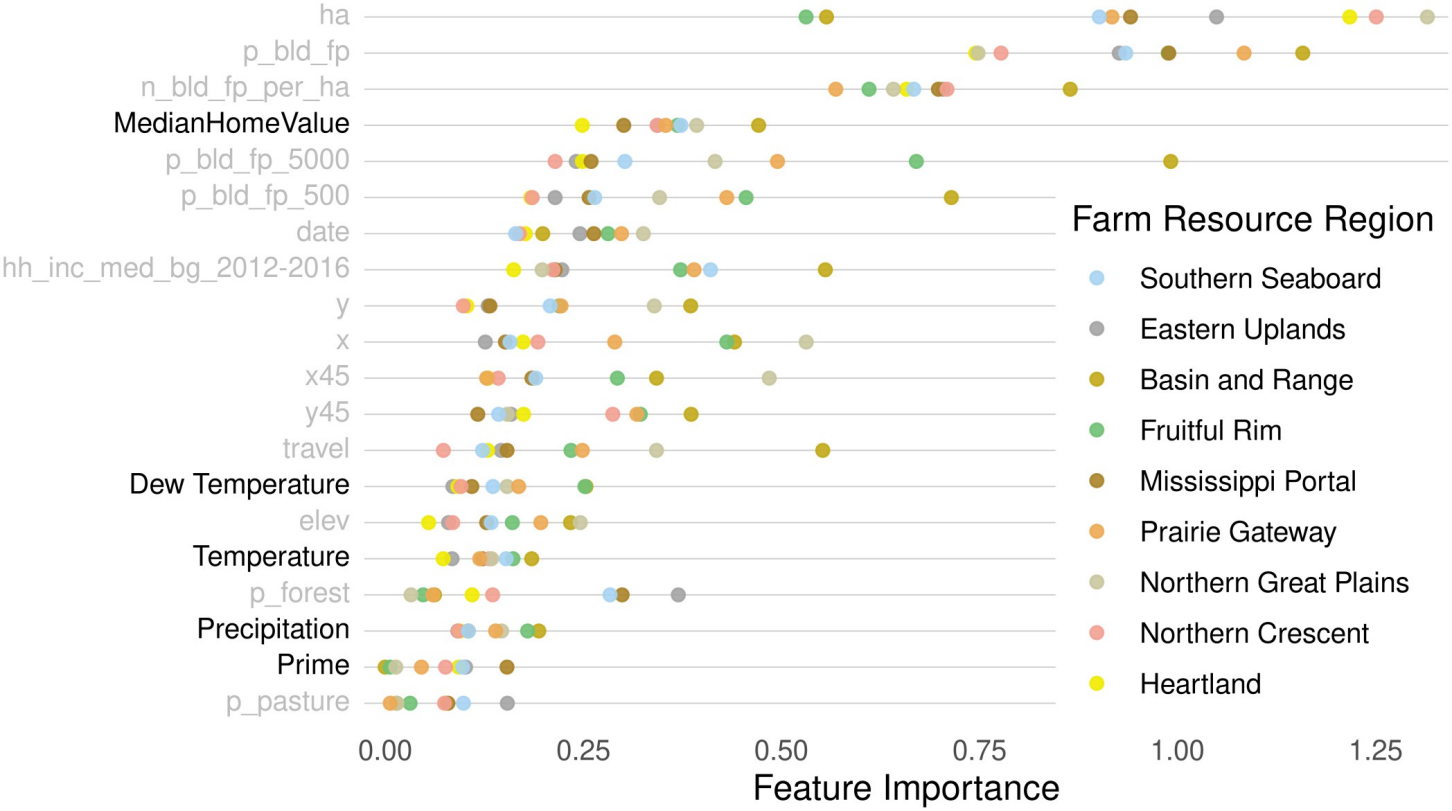
Permutation feature importance, farm resource region. Dot plots of permutation feature importance for the top 20 predictors, across all farm resource region-level models using the full predictor set. Predictors added by this paper are denoted in bold, and climatic variables have been grouped for clarity. Each dot represents a farm resource region model.

Individually and collectively, the additional predictors in our “Full” set offer modest and meaningful improvements to model performance relative to the “Restricted” set. Figs 5 and 6 show the relative predictive contributions of each feature in the Full predictor set for models estimated at the county and farm resource region level, respectively. The most influential additions of this paper are the climatic variables (precipitation, dew temperature, and temperature) and real estate indicators (house price index at the county level and median home value at the farm resource region level), followed by prime soil and soil of statewide importance, which is one quality tier below prime and does not appear in the top 20 features depicted in Figs 5 and 6. Irrigation status, both ever-irrigated and irrigated in the three years preceding the parcel’s sale, contribute little predictive power, although both exhibit significant heterogeneity across counties. Conditionally prime soil types (prime if warm enough, if protected, if irrigated, etc.) and soil types important only at local scales offer little prediction importance to the model. See S2 Fig for the importance of all features used as model predictors.

**Fig 7:**
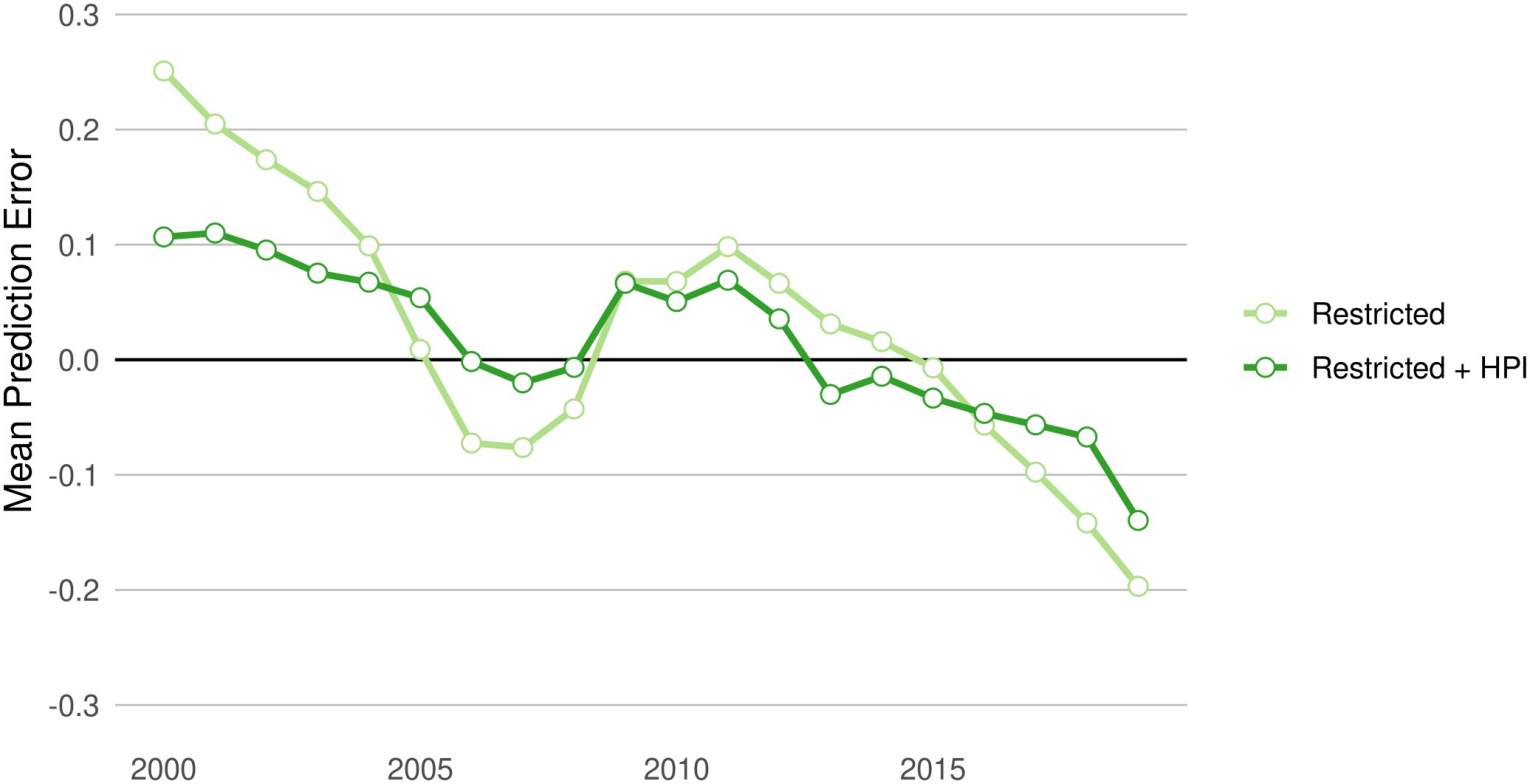
The addition of HPI attenuates model error trend. Mean annual prediction error in Restricted county model, compared to county level models estimated with Restricted predictor set plus the annual, county-level housing price index.

Accounting for changes in related real estate markets plays an especially important role in better predicting fair market value. Fig 7 shows the effect of adding housing price index (HPI) data to the otherwise restricted set of predictors in the county-level model. While over-time heteroskedasticity remains even after the addition of HPI, its inclusion reduces the magnitude of the error, particularly the over-prediction in the early years of the sample and the under-prediction in its later years.

**Fig 8:**
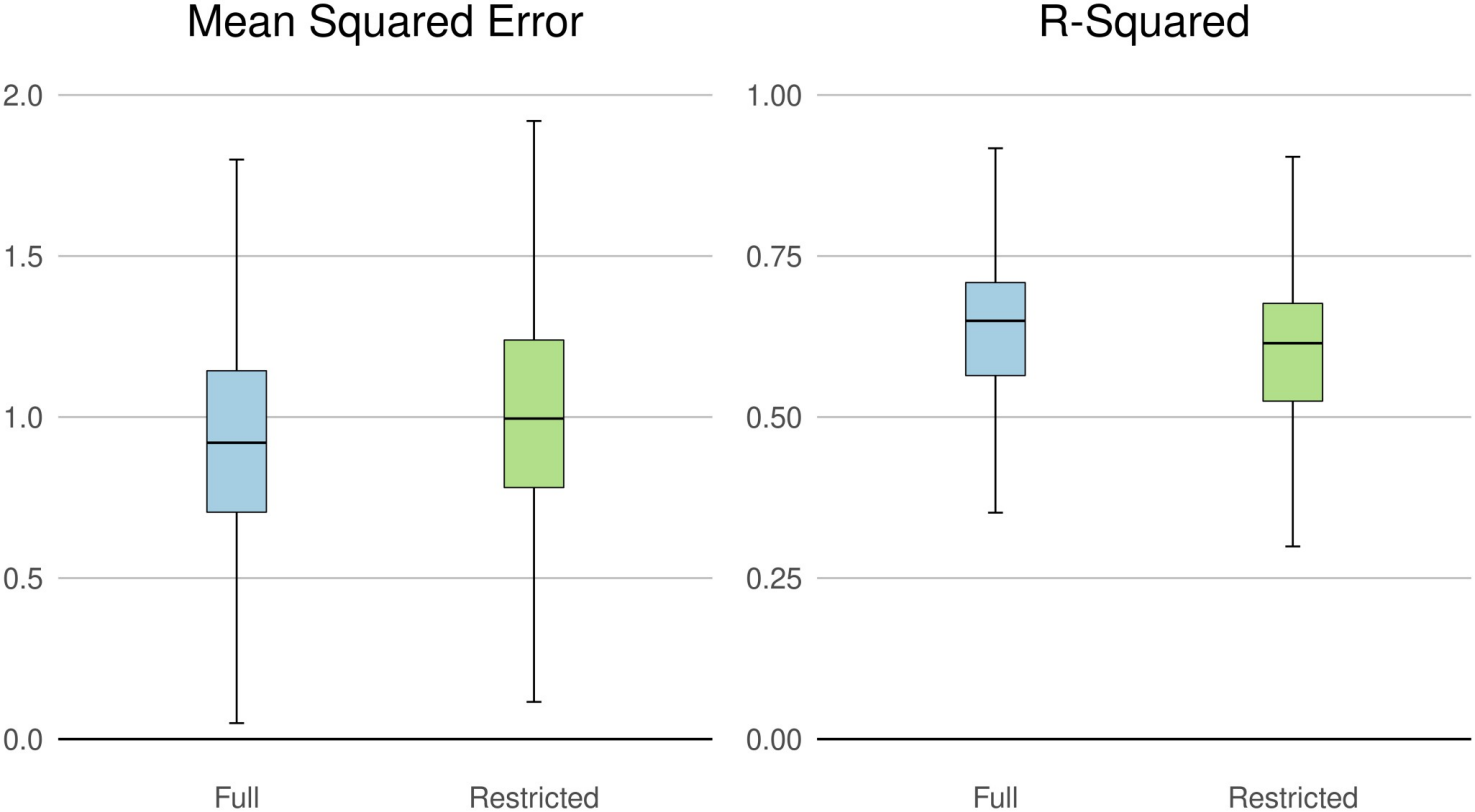
Full vs. Restricted predictor county-level model performance. A box plot comparison of model performance (MSE and *R*^2^) with the restricted (green) and full (blue) predictor sets at the county level. Outliers removed from visualization for clarity.

Altogether, our Full model offers modest but consistent improvements in model performance at the county level. Fig 8 shows the average county-level mean squared error and r-squared of the Full and Restricted models. Nationally, the Full model outperforms the Restricted model, with an average mean squared error (in logged 2020 USD per hectare) of 0.93 and an average R-squared of 0.65 compared to 1.02 and 0.61 respectively, in the Restricted model.

### The effect of regional scale modeling

**Fig 9:**
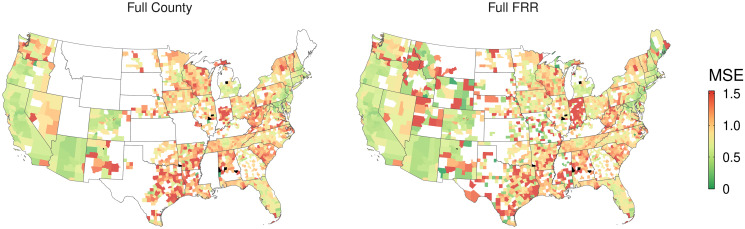
Spatial comparison of coverage and accuracy of county and FRR models. Maps display the mean squared error of parcel-level predictions, by county, for county-level models (left) and farm resource region models (right) using the Full predictor set. To preserve meaningful color variation, the top of the color gradient corresponds to the 90th percentile of MSE values, and counties with outlier values have been visually censored to that value. Blacked-out counties indicate instances wherein a county model was specified but the farm resource region model did not include testing observations in that county.

Specifying models at the scale of the farm resource region enhances accuracy and enables FMV predictions for parcels in counties that, even after drawing on observations from neighbors, do not meet the minimum observation threshold for county-level modeling. In Fig 9, we see that many states which are wholly absent from the county models receive predicted land values in the farm resource region model, including Montana, Maine, Idaho, Wyoming, Mississippi, and Kansas. Several other states see their coverage greatly extended. In Missouri, for instance, the county models are only able to generate land value estimates in the urban and exurban regions surrounding St. Louis. The farm resource region models, by contrast, expand into the rural hinterland of Missouri. Overall, farm resource region modeling extends coverage by 376 counties, constituting an additional area of 1.35 million km^2^ (the added properties make up 818.7 km^2^). Across these added counties, model performance remains well within the previously observed range, with a median mean squared error of 0.92 and a mean of 1.53.

Elsewhere, we see improvements to predictive accuracy. In counties with fewer than 1000 sales observations (before drawing on observations from neighbors), the farm resource region model outperforms the county model (*MSE* = 1.02 versus *MSE* = 1.07 using the Full predictor set). The benefits of modeling at the FRR scale remain even at higher observation densities. In counties with greater than 1,000 observations, the FRR model with the Full predictor set produces an average mean squared of error of 0.75, compared to 0.77 from the county model.

**Fig 10:**
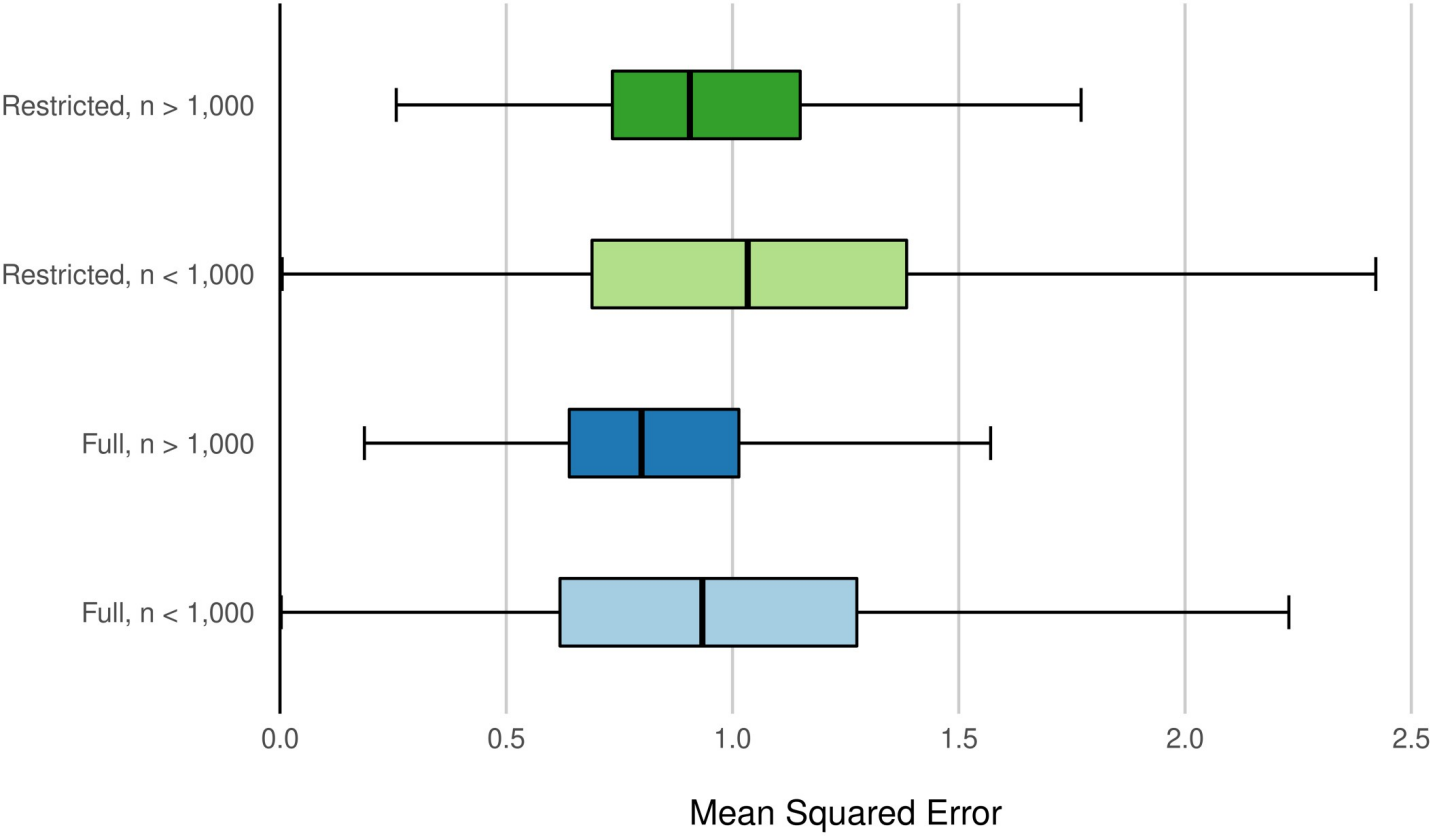
MSE by size of sample and predictor set. Box plots of average county-level MSE from farm resource region models. Box plots for models estimated with the Full predictor set are in blue, while those estimated with the Restricted set are in green. Box plots for the group of counties with less than 1,000 observations have lighter shading than plots for the group counties with greater than 1,000 observations. Outliers removed from visualization for clarity.

The benefits of the Full predictor set remain in regional-scale modeling. Across both county-observation size categories, the Full model outperforms the Restricted model (Fig 10). Considering the set of parcels for which data allow modeling under both the Restricted and Full predictor sets, modeling at the FRR scale using the Full set delivers a median mean squared error of 0.76 compared to 0.87 for the Restricted set. This improvement is comparable to that observed for county-scale models, where using the Full set results in a median mean squared error of 0.93, compared to the Restricted model’s 1.02 median mean squared error.

Features added in the Full model contribute to predictive power in similar patterns to feature importance across county models. Fig 6 shows the distribution of feature importance by farm resource region. Median home value is the fourth most important predictor, after parcel size and several building characteristics. Climatic variables continue to offer substantial importance, with dew temperature, temperature, and precipitation falling in the upper half of all predictors. As in the county models, prime soil and soil of statewide importance remain considerably higher ranked than all conditional soil types, which consistently offer near-zero feature importance across all nine regions. Though it is not among the top 20 features displayed in Fig 6, irrigation status exhibits modest feature importance across both ever-irrigated and irrigated in the last three years (S1 and S2 Figs).

Taken together, the added predictors and regional scale modeling improve overall accuracy by approximately 15 percent. When compared across common parcels, the farm resource region model with the full predictor set produces predictions with an average mean squared error of 0.96, compared to the county model using the restricted set of predictors, which produces a mean squared error of 1.11, on average.

## Discussion

### Accuracy improvement mechanisms

Both at the county and farm resource region levels, the Full set of predictors improve model performance. The mechanism through which this improvement acts is likely straightforward. The predictors added by this study contribute valuable information to the tree-based ensemble model, allowing it to more accurately “bin” observations. That climate and soil characteristics should help predict land value is consistent with the theoretical foundations and empirical results of the Ricardian land attribute capitalization literature. In contrast, our real estate market indicators, house price index (county models) and median home value (farm resource region models), capture broader market dynamics rather than capitalized attribute values. The consistent feature importance exhibited by both real estate predictors suggests that having access to high quality indicators of spatiotemporal variation in local and regional residential real estate markets may greatly improve conservation planners’ ability to estimate the cost of non-residential properties, including the vacant, undeveloped, and agricultural parcels of interest to the present study.

Regardless of predictor set, we observe that the farm resource region models improve model performance, relative to county modeling. Enlarging the geographic scale of modeling allows for the incorporation of more data points. The improvement in performance is likely due to the greater number of observations on which the models draw, consistent with sample size-performance relationships observed in county-level modeling in Fig 1. In the FRR models, we observe disproportionate accuracy improvements for sales value predictions of parcels in counties with under 1000 observations (Fig 10), where MSE improves by 6% compared to 2% in counties with greater than 1000 observations. Sales observations in low-density North Dakota, for example, that would otherwise have been siloed off with only a handful of other observations in their respective counties (or discarded altogether) are now modeled alongside observations in segments of Wyoming, Montana, and Minnesota, among other states in the Northern Great Plains region.

However, having more observations does not guarantee better performance. The predictive benefit of more observations will not always outweigh the predictive cost of pooling parcels across areas that could exhibit fundamentally different relationships among variables. That tension could explain the relatively similar performance of our models across regions of very different sizes (S3 Fig). Take for example the Fruitful Rim and Great Plains regions. The first has vastly more observations, but is spread across areas with very different land markets. Conversely, in the example of low-density North Dakota above, the contiguous nature of its farm resource region allows observations to be modeled alongside observations that share agricultural and climatic characteristics, as well as commonalities among other determinants of land value, such as local non-agricultural land markets. The trade-off between the number of observations and their commensurability highlights the importance of the choice of spatial model boundaries and the value of farm resource regions as a modeling domain.

### Coverage benefits

The broader coverage afforded by our farm resource region modeling allows for conservation planning in areas that would otherwise need to rely on cruder estimates. For instance, across the entire state of Montana, our sample contains only 507 observations, all of which are discarded under the county-level modeling paradigm. Farm resource region models allow for such observations to be grouped and modeled alongside observations in either the Basin and Range or Northern Great Plains which bear a resemblance to those otherwise discarded Montana observations. In the absence of the farm resource region approach, policy analysts and conservation planners would be forced to use lower-resolution estimates, which have been shown to lead to substantial mischaracterization of cost-effective conservation strategies (Nolte 2020). The expansion of model predictions into rural areas afforded by the farm resource region models (see, for example, the expanded coverage in Missouri in Fig 9) brings high-resolution land value estimates to areas that might offer important opportunities for high-impact conservation easements and fee simple purchasing.

### Model improvements in practice

To assess the practical benefits of our modeling approach, one might ask whether it facilitates more cost-effective achievement of fixed conservation goals. Agencies require accurate estimates of the minimum cost necessary to achieve a set conservation target. For instance, the Biden administration has established the ambitious target of conserving 30 percent of US lands by 2030 [16]. At least a portion will be secured via financial contracting with private landowners. How much spending the project requires is largely unknown prior to contracting, motivating the use of predictive modeling to inform implementation. As a demonstrative exercise, we imagine a single agency is tasked with purchasing 30 percent of the land in our universe of observed sales, equipped with a set of land value predictions alternatively generated by: a) the true underlying sales value; b) the full-predictor farm resource region model; or c) the restricted-predictor county model. Using each of these approaches, we rank the entire universe of sales records by ascending predicted price, designating parcels for purchase until 30 percent of the total land area (8.4 million hectares) has been conserved.

The farm resource region model offers the potential for greater cost-effectiveness through two channels: improved accuracy or inclusion of lower-cost parcels by virtue of expanded coverage. If the total conservation target increased proportionally with the size of the candidate parcel set, expanded coverage would only offer an advantage if the distribution of prices in the newly included areas were systematically cheaper. In this case, we hold the target constant, meaning expansion offers an advantage even where the underlying price distribution remains the same. To disentangle the mechanisms by which expansion improves cost-effectiveness, we also create candidate parcel sets for the true information and full resource region model that only include parcels for which the restricted county model can generate predictions.

**Fig 11:**
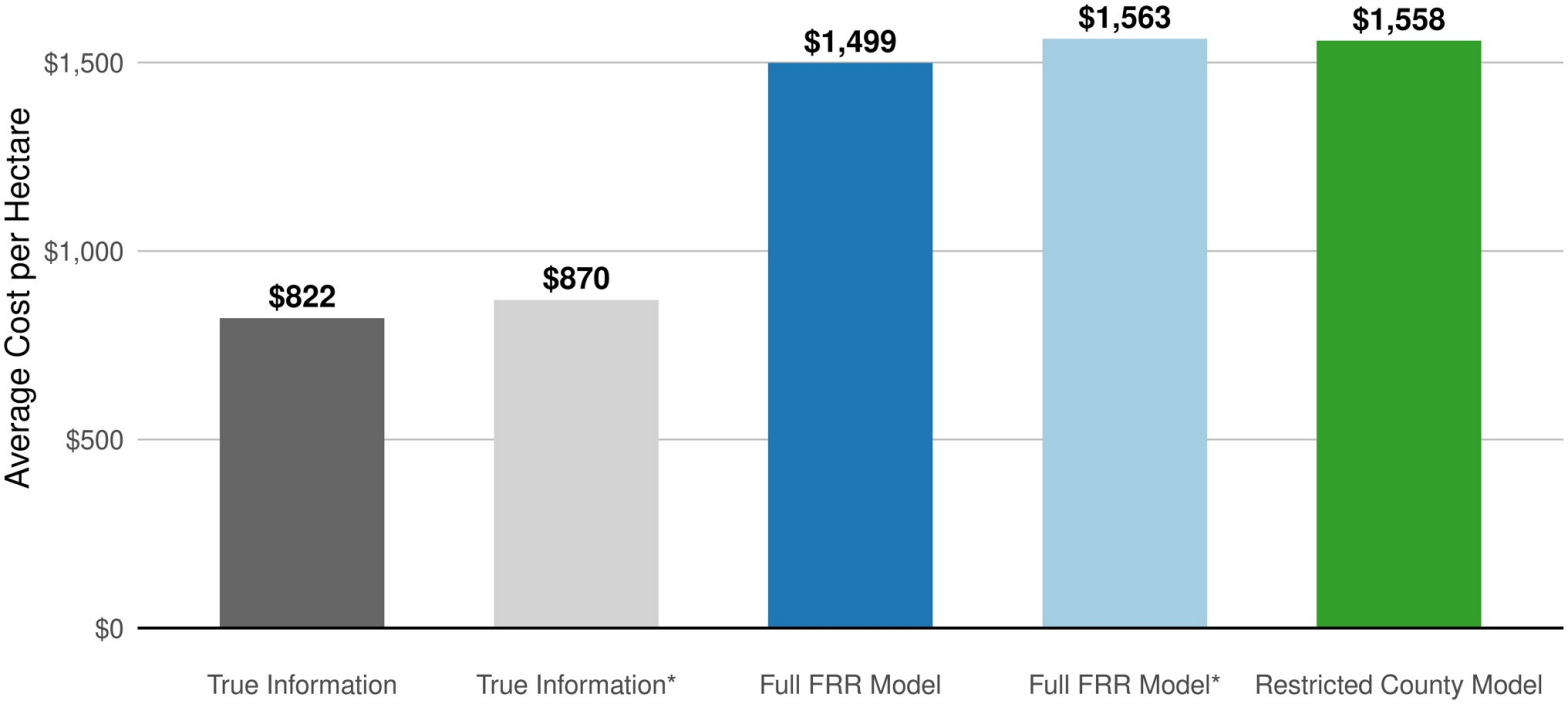
30x30 average cost per hectare. A bar chart of costs calculated on the basis of the inflation-adjusted, observed sale values of parcels hypothetically purchased to conserve 30 percent of the land area represented in our sales data. Purchase ordering is determined by the predicted sales prices generated by each model. Costs for purchase orderings based on actual sales data are in gray; costs based on FRR-level predictions in blue; and costs based on county-level models in green. Asterisks (and bars with lighter shading) denote purchase simulations in which the set of candidate parcels has been restricted to match the set for which county-level models have sufficient data to generate predictions.

Our simulation results suggest a meaningful economic benefit from improvements in model coverage. Fig 11 compares average expenditures per hectare across models. Where the candidate sets are identical (denoted by an asterisk in Fig 11), the full resource region model and the restricted county model produce nearly identical average costs, suggesting the savings arise from the expanded set of predictions from which the agency can select candidates for purchase. Working with the unrestricted candidate set, if the agency had access to the true underlying sale values, the endeavor would cost $822 per hectare. Using the restricted-predictor county model to target purchasing would nearly double the cost, to $1,558 per hectare. Relative to this, the full-predictor resource region model could save the agency $60 per hectare, about 3.85 percent.

Using the average per-hectare costs, we estimate costs in a more realistic “30x30” conservation scenario. After excluding already protected land, an agency would still need to conserve approximately 172 million hectares to protect 30 percent of US land area [17]. Scaling our average per-hectare costs by the target land area, we find that, employing the full-predictor resource region model to target contracting, the agency would expend $258 billion dollars, $10 billion less than would be spent if the restricted-predictor county model were utilized. The annual “rental” value of that savings, calculated at 1.5 percent interest (approximately the long-term real rate on the U.S. Treasury’s inflation protected securities), is $15 million. For context, the USDA annually spends just under $500 million on land easements (e.g., Agricultural Conservation Easement Program) and between $2 and $2.5 billion on land retirement (e.g., Conservation Reserve Program) [18].

## Conclusion

To protect critical habitats and ecosystems, conservation advocates, policymakers, and managers rely on land cost estimates to weigh competing strategies in the face of scarce resources. Hindered by limitations of data and computing power, past efforts to produce land value estimates have suffered from low spatial resolution. Nolte (2020), approaching the general problem of land value estimation, established a methodology and database that greatly improves value estimates of undeveloped land, employing parcel-level sales data and a large set of predictors. As developers and agricultural producers look to undeveloped or partially agricultural land for conversion to lucrative but environmentally damaging use, conservation planners require a thorough and reliable picture of property valuation in order to cost-effectively build conservation agendas.

In this paper, we improve the accuracy and coverage of previous estimation models for undeveloped land value, leveraging an added set of economically-relevant, high resolution climatic and ecological predictors, as well as incorporating time series data on county-specific residential housing markets. Modeling at larger scales that correspond to regional agricultural markets expands spatial coverage and accuracy, offering a new standard for applications and further improvements.

We test our modeling approach using one such exemplary application: the ambitious target of protecting 30 percent of US lands by 2030. Using the improved modeling methods introduced here, we find that an implementing agency could save approximately $15 million annually–around 4 percent of the Agricultural Conservation Easement Program’s annual budget–from more cost-effective targeting of land purchases.

## Supporting information

S1 Fig Permutation feature importance from full county model, all features. Climatic variables grouped for clarity. Features added by this paper denoted in bold. Each dot represents a single county.
(TIF)

S2 Fig Permutation feature importance by farm resource region, all features. Climatic variables grouped for clarity. Features added by this paper denoted in bold.
(TIF)

S3 Fig Prediction accuracy and sample size by farm resource region. Prediction accuracy varies little across farm resource regions and shows no apparent correlation with the number of observations in a region.
(TIF)

S1 Appendix Parcel filter. (DOCX)

S2 Appendix Multi-parcel aggregation. (DOCX)
