# Supplementary figures and images for: Expanding the coverage and accuracy of parcel-level land value estimates
Source: PLoS One. 2023 Sep 8;18(9):e0291182. doi: 10.1371/journal.pone.0291182 (PMC10490921; doi:10.1371/journal.pone.0291182)

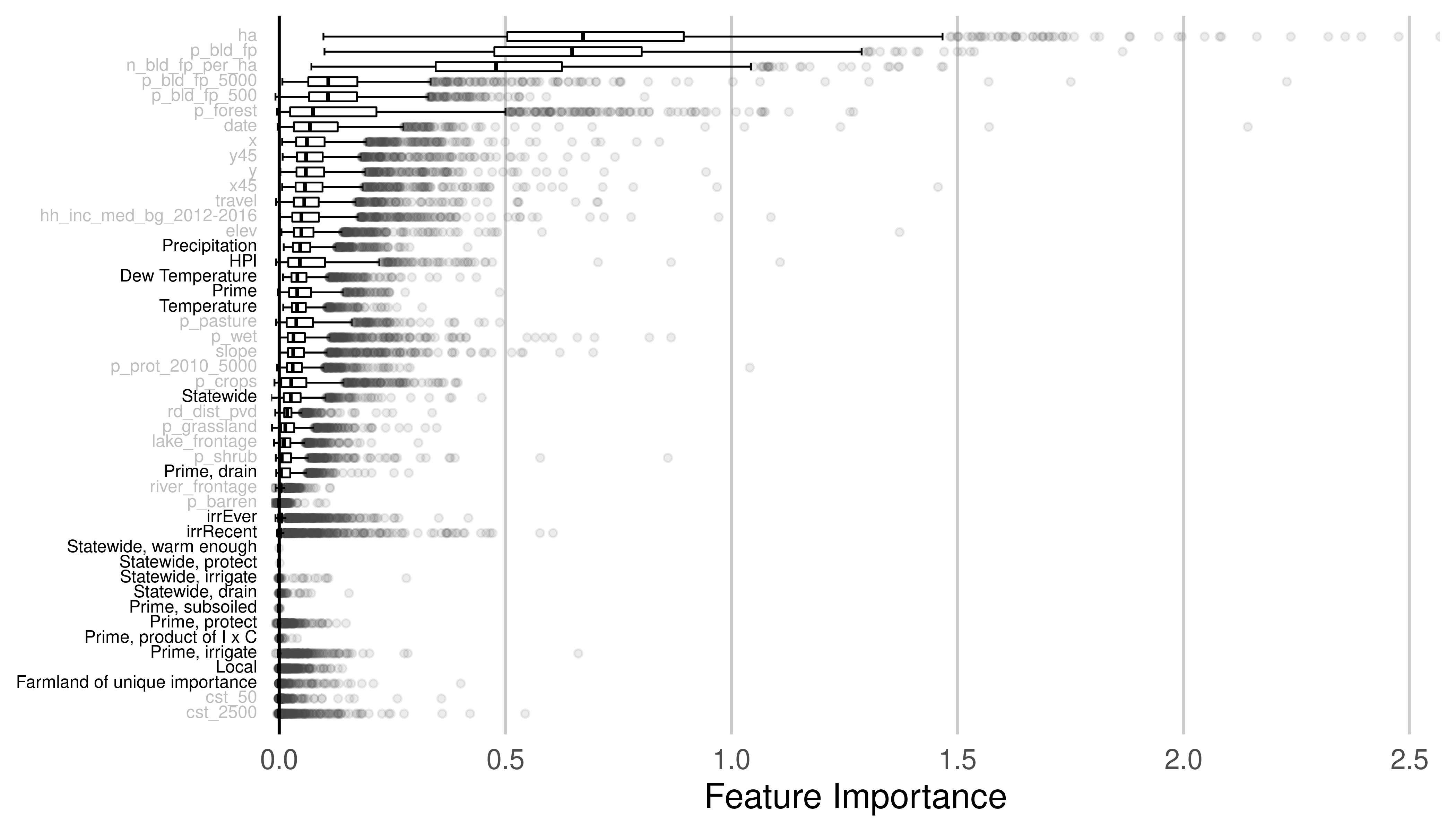

Supplement: S1 Fig — Climatic variables grouped for clarity. Features added by this paper denoted in bold. Each dot represents a single county. (TIF) [file pone.0291182.s001.tif]

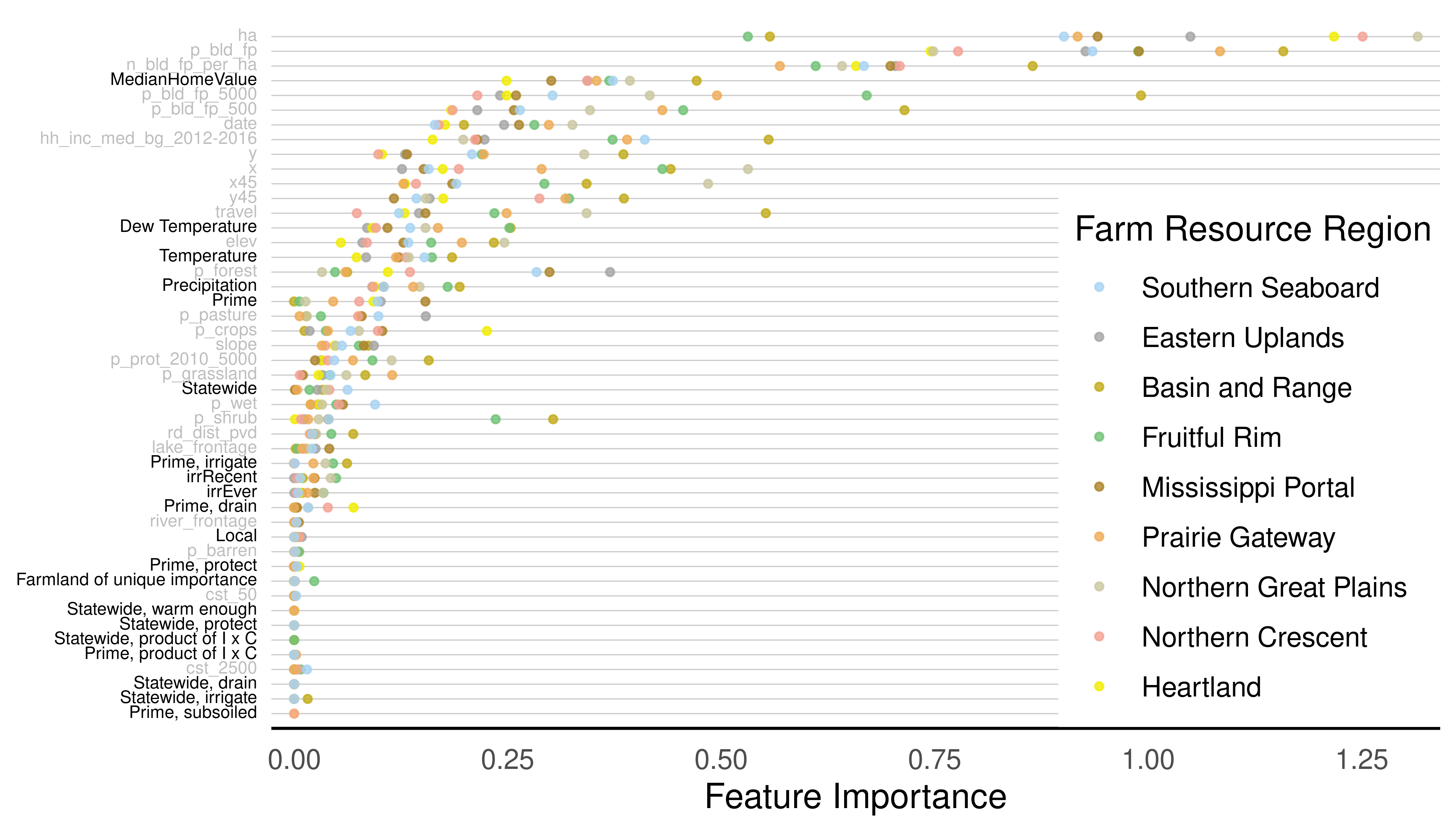

Supplement: S2 Fig — Climatic variables grouped for clarity. Features added by this paper denoted in bold. (TIF) [file pone.0291182.s002.tif]

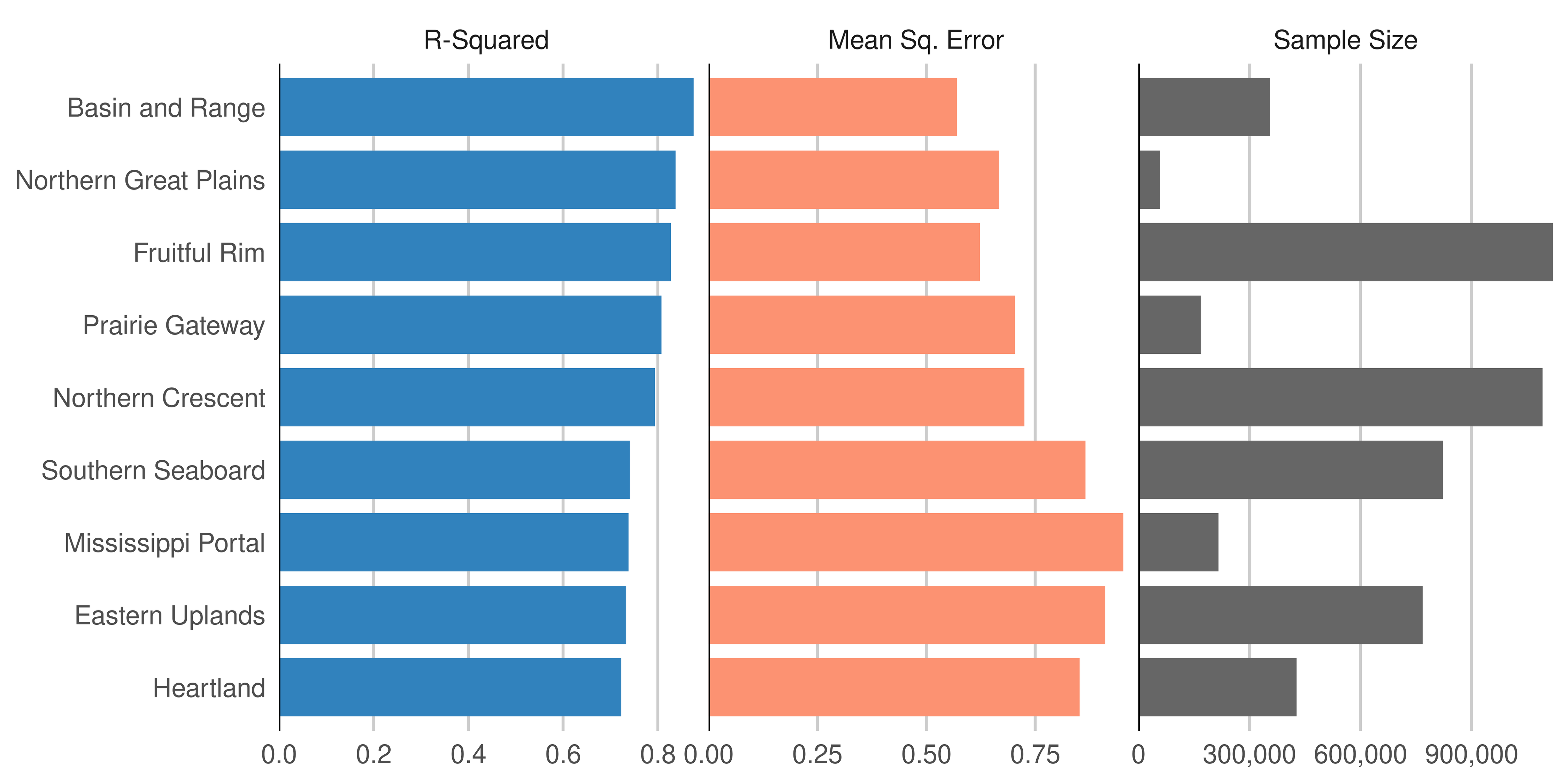

Supplement: S3 Fig — Prediction accuracy varies little across farm resource regions and shows no apparent correlation with the number of observations in a region. (TIF) [file pone.0291182.s003.tif]
