# Supplementary material for: Expanding the coverage and accuracy of parcel-level land value estimates
Source: PLoS One. 2023 Sep 8;18(9):e0291182. doi: 10.1371/journal.pone.0291182 (PMC10490921; doi:10.1371/journal.pone.0291182)
Supplement: S1 Appendix — (DOCX) [file pone.0291182.s004.docx]

S1 Appendix.

Parcel selection filter:

1. Meets “undeveloped” criteria established by Nolte (2020), or
2. Over half its area was classified as either grassland/herbaceous or
3. Planted/cultivated in the 2011 National Land Cover Database (NLCD), or
4. Over half its area was classified as agricultural in the 2000 Land Change Monitoring, Assessment, and Projection (LCMAP), or
5. Had any of the following ZTRAX land use codes:
   1. Agricultural (“AG”);
   2. Homestead (“MS113”);
   3. Vacant open space/conservation/forest land (“VL104”);
   4. Vacant agricultural/unimproved (“VL108”)
