# Supplementary material for: Expanding the coverage and accuracy of parcel-level land value estimates
Source: PLoS One. 2023 Sep 8;18(9):e0291182. doi: 10.1371/journal.pone.0291182 (PMC10490921; doi:10.1371/journal.pone.0291182)
Supplement: S2 Appendix — (DOCX) [file pone.0291182.s005.docx]

S5 Appendix.

Multi-parcel Aggregation.

Our analysis employs sales as the individual sample unit. In the case of sales involving multiple parcels, we aggregate predictor values across sales. We take the arithmetic mean of all Nolte (2020) variables except river and lake frontage length, which we sum across all parcels. For all climatic variables added by this paper (precipitation, dew temperature, and temperature), we take the area-weighted mean across all parcels. For irrigation indicator variables (ever irrigated and irrigated in the past three years), we take the maximum of all parcels’ indicators. If any parcel in the sale has been irrigated, the sale is considered irrigated. For soil types, we calculate a given soil type *j*’s proportion *p_j_* as the proportion of total soil area across each parcel *i* occupied by that soil type:

$$p_{j}= \frac{\sum_{i=1}^{n} {area}_{j,i}}{\sum_{i=1}^{n} {area}_{i}}$$
